# Uterine and Cervical Cancer in Iran: An epidemiologic analysis of the Iranian National Population-Based Cancer Registry

**DOI:** 10.34172/aim.2023.01

**Published:** 2023-01-01

**Authors:** Hani AziziKia, Hamidreza Didar, Azin Teymourzadeh, Amin Nakhostin-Ansari, Pooya Jafari Doudaran, Bahareh Ferasatifar, Armin Hoveidaei, Gholamreza Roshandel

**Affiliations:** ^1^Student Research Committee, School of Medicine, Shahroud University of Medical Sciences, Shahroud, Iran; ^2^Preventative Gynecology Research Center, Shahid Beheshti University of Medical Sciences, Tehran, Iran; ^3^Imam Khomeini Hospital Complex, Tehran University of Medical Sciences, Tehran, Iran; ^4^Sports Medicine Research Center, Neuroscience Institute, Tehran University of Medical Sciences, Tehran, Iran; ^5^Faculty of Medicine, Qom University of Medical Sciences, Qom, Iran; ^6^Department of Chemistry, Iran University of Science and Technology, Tehran, Iran; ^7^BSc in Radiotherapy Technology, Student Research Committee, School of Allied Medical Sciences, Shahid Beheshti University of Medical Sciences, Tehran, Iran; ^8^Golestan Research Center of Gastroenterology and Hepatology, Golestan University of Medical Sciences, Gorgan, Iran

**Keywords:** Epidemiology, Uterine cervical neoplasms, Uterine neoplasms

## Abstract

**Background:**

Gynecologic cancers, including neoplasms of the cervix and uterine, are the fourth most common malignancies, causing 3.46% of deaths in women aged 15 to 59.

**Objectives:**

We aimed to report the Iranian National Population-based Cancer Registry (INPCR) results for Cervical and Uterine cancers in 2017.

**Methods:**

The total population of Iran in 2017 was 80881792. INPCR collected data on cervical and uterine cancer incidence from 31 provinces of Iran. In this project, we retrospectively examined all the country’s regions in terms of screening for the existence of these two cancers. The registry data bank in Iran was used.

**Results:**

Overall, 3481 new cervical and uterine cancer cases were registered in INPCR, including 842 cases of cervical cancer (with a crude rate of 1.04) and 2639 cases of uterine cancer (with a crude rate of 3.26). The average age-standardized incidence rate (ASR) was 0.99 for cervical cancer and 3.29 for uterine cancer. Out of 3481 new cervical and uterine cancer cases, 2887 were registered with pathological findings and 594 without pathological confirmation. In cervical cancers, the highest rate was related to squamous cell carcinoma, with 486 cases (57.72%).

**Conclusion:**

Our results showed that Iran is a low-risk area for the incidence of cervical and uterine cancers. In this study, the highest rate of cervical cancer was related to squamous cell carcinoma, confirming previous reports. However, this rate was lower than previous studies and suggested an increase in other types of cervical cancer in Iran.

## Introduction

 Gynecologic cancers, including neoplasms of the cervix and uterine, are the fourth most common malignancies, involving approximately 499 727 women worldwide in 2016, and causing 3.46% of deaths in women aged 15 to 59.^1^

 Cervical cancer is the fourth most common cancer and the third leading cause of death due to cancer in women.^1^ Cervical cancer is one of the leading causes of death in women in some parts of the world, such as Africa and South Asia.^2,3^ Each year, 528 000 new cases of cervical cancer are diagnosed worldwide, of which 200 000 end in death.^3-5^ Cervical cancer occurs at younger ages compared to breast, lung, and ovarian cancers.^2^

 Uterine corpus cancer is the sixth most commonly diagnosed cancer in women, with 417 000 new cases and 97 000 deaths in 2020.^6^ The prevalence of uterine cancer in Iran has increased threateningly over the past two decades.^7^ According to previous studies, Iran will have a significant increase in gynecologic cancer incidence and mortality in the coming decades, especially in the central regions.^5^ There have been several reports of cancer incidence and mortality in Iran recently. The age-standardized incidence rate (ASIR) and the age-standardized mortality rate (ASMR) for uterine cancer in Iran are 2.14 and 0.93 per 100 000 people.^8^ In Iran, the incidence of gynecological cancers increased from 2.5 per 100 000 in 1990 to 12.3 per 100 000 in 2016, and the most common cancer among women changed from cervical cancer in 1990 to uterine cancer in 2016.^9^ Some studies in Iran demonstrated that uterine cancer had been the most common cancer among women in recent years. The mean age of diagnosis was increased in patients with ovarian cancer and decreased in patients with cervical cancer.^10^

## Objectives

 This study aims to assess the incidence and investigate the prevalence of cervical cancer and uterine cancer based on the Iranian National Population-Based Cancer Registry (INPCR).

## Material and Methods

###  Study Design

 In this study, all new cases of uterine and cervical cancers in Iran in 2017 were included and added to the study’s sample. Data on cancer patients were obtained from the INPCR secretariat in the Ministry of Health. Then, the population statistics of Iran in 2017 by provinces were received through the Statistics and Information Technology Management Center of the Ministry of Health and Medical Education. The required information was referred to obtain information from patients via email taken by the Ministry of Health from the laboratory centers where the pathology samples of these patients were examined. This information included demographic characteristics, pathological characteristics of the disease, and time of laboratory cancer diagnosis. Also, the lesion site, degree of differentiation and the degree of tumor invasion were recorded in the files. To ensure that the data was correct, patient forms were stored in the final form when they filled out the data. This saving of the form reduced the possibility of data interference and exchange. In this study, pathological and demographic information was recorded about new primary tumors that had malignant behavior. In case of metastasis or recurrence, only primary tumors were recorded. Then, the data set was examined in terms of possible duplicate records. Finally, the data were analyzed in terms of number, percentage, crude incidence rate, age-specific incidence rate, standardized age incidence rate (ASR) at the provincial and national levels. ASRs were calculated using the CanReg5 software, and rates were expressed per 100 000 populations.

###  Statistical Analysis

 The Statistical Package of Social Science Software (SPSS version 23, IBM Company, USA) was used to analyze the data. Continuous variables are shown as mean (standard deviation) or median [interquartile range (IQR)], and categorical variables are demonstrated as frequency (percentage).

## Results

 In 2017, INPCR recorded 3481 new cervical and uterine cancer cases, including 842 cases of cervical cancer, with a crude incidence rate of 1.04, and 2639 cases of uterine cancer, with a crude incidence rate of 3.26. The ASR was 0.99 and 3.29 for cervical and uterine cancer, respectively.

**Figure 1:**
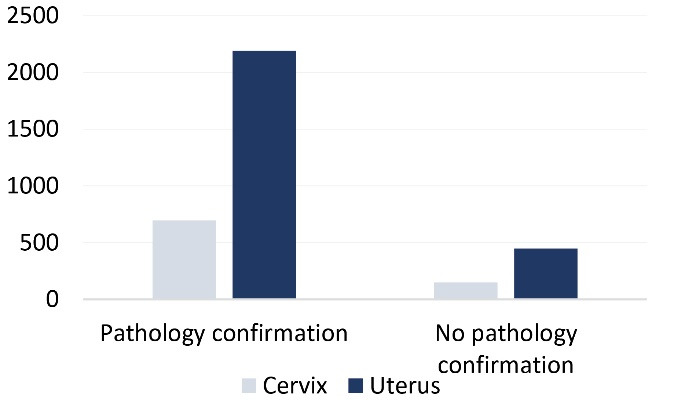
Incidence of Cervical Cancer and Uterine Cancer Based on the Diagnostic Method

 Among the reported cases of cervical cancer, 695 patients (82.54%) were registered with pathological confirmation and 147 (17.46%) without any notes. For uterine cancer, 2192 cases (83.06%) were confirmed by pathological findings, and 447 patients (16.94%) were reported without pathological confirmation (Figure 1).

Table 1 summarizes the incidence and ASR of cervical cancer in different provinces. Hormozgan, Yazd, Golestan, and Semnan had the highest ASRs (ASR ≥ 1.5), while Bushehr, Ghazvin, Kohkilooyeh and Boyer-Ahmad, Sistan and Baloochestan, and Fars had the lowest ASRs (ASR ≤ 0.5).

**Table 1:** Incidence and ASR of Cervical Cancer in Different Provinces

**Province**	**Number**	**ASR**
Hormozgan	25	1.77
Yazd	17	1.74
Golestan	29	1.59
Semnan	12	1.50
East Azarbayjan	68	1.48
West Azarbayjan	46	1.38
Razavi Khorasan	84	1.33
Khoozestan	52	1.32
Ardabil	15	1.14
Guilan	38	1.14
Isfahan	64	1.06
Mazandaran	42	1.03
Kerman	29	1.01
Tehran	161	1.00
North Khorasan	8	0.93
Alborz	24	0.76
Chaharmahal Bakhteiari	7	0.73
South Khorasan	6	0.73
Qom	9	0.69
Hamedan	13	0.69
Ilam	4	0.63
Zanjan	7	0.63
Kermanshah	13	0.63
Markazi	11	0.63
Kordestan	10	0.61
Lorestan	8	0.52
Fars	26	0.50
Sistan Baloochestan	6	0.38
Kohkilooye Boyerahmad	2	0.31
Ghazvin	4	0.25
Busher	2	0.24
**Overall **	**842**	**0.99**

ASR, age-standardized incidence rate.

Table 2 shows the incidence and ASR of uterine cancer in different provinces of Iran. According to the results, the ASR of uterine cancer was the highest in Razavi Khorasan, Yazd, and North Khorasan (ASR ≥ 4), while it was the lowest in Kohkilooyeh and Boyer-Ahmad, Sistan and Baloochestan, Lorestan, and Zanjan (ASR ≤ 1).

**Table 2:** Incidence and ASR of Uterine Cancer in Different Provinces of Iran

**Province**	**Number**	**ASR**
Razavi Khorasan	456	6.56
Yazd	50	5.12
North Khorasan	37	4.01
Tehran	581	3.96
Isfahan	206	3.80
Mazandaran	145	3.71
Khoozestan	138	3.69
Guilan	118	3.60
Semnan	27	3.56
Fars	169	3.55
Qom	35	3.26
East Azarbayjan	131	3.04
Kerman	80	3.01
Alborz	76	2.78
Golestan	44	2.74
Ilam	13	2.47
Hormozgan	26	2.26
Hamedan	39	2.13
West Azarbayjan	61	2.05
South.Khorasan	15	1.86
Markazi	27	1.85
Ardabil	22	1.74
Kermanshah	34	1.70
Kordestan	23	1.49
Chaharmahal Bakhteiari	12	1.40
Ghazvin	16	1.38
Busher	13	1.36
Zanjan	10	1.00
Lorestan	15	0.97
Sistan Baloochestan	16	0.94
Kohkilooye Boyerahmad	4	0.67
**Overall**	**2639**	**3.29**

ASR, age-standardized incidence rate.

**Figure 2:**
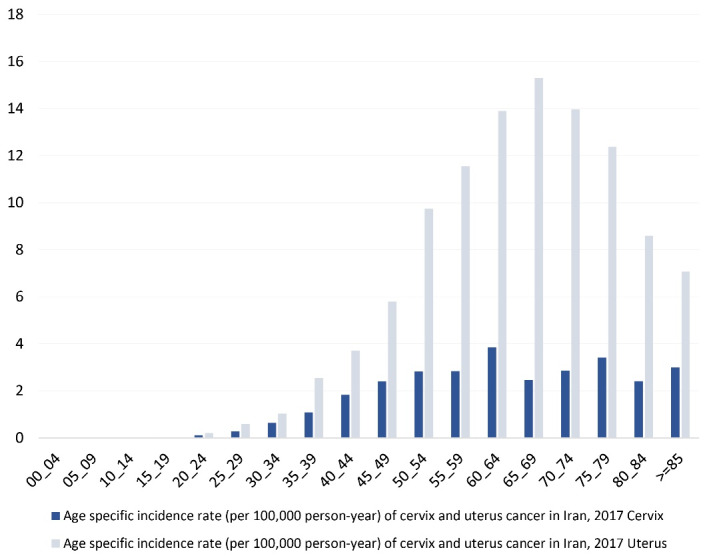
Age-Specific Incidence Rate (Per 100 000 Person-Year) of Cervical and Uterine Cancer in Iran, 2017

 The highest incidence rate of cervical cancer pertained to females aged 60–64 years (3.85), and it gradually decreased with age until it increased again in those aged 75–79 years (3.41). Regarding uterine cancer, the highest incidence rate was found in women aged 65 to 69; it gradually decreased as they advanced in age (Figure 2).

**Figure 3:**
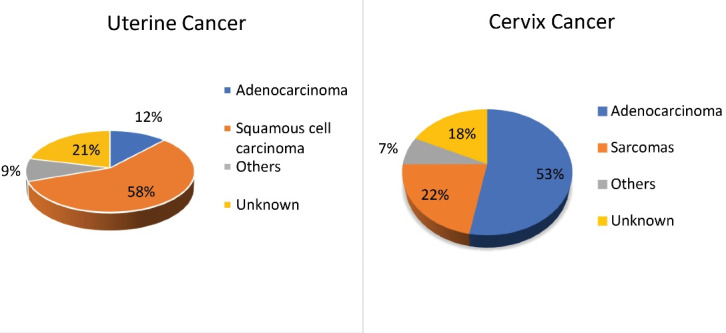
Frequency of Cervical and Uterine Cancer in Iran in 2017, by Histology

 Squamous cell carcinoma accounted for most cases of cervical cancer (57.72%), while adenocarcinoma accounted for the majority of cases of uterine cancer (52.97%) (Figure 3).

 Tables S1 and S2 (see Supplementary file 1) show the histological types of cervical and uterine cancers found in different provinces. The incidence of cervical squamous cell carcinoma in Bushehr, Ghazvin, Kohkilooyeh and Boyer-Ahmad was higher than other provinces. The incidence of uterine adenocarcinoma was greater in Ghazvin, Golestan, Yazd, and Zanjan provinces than other provinces (more than 70% of cases). The percentage of uterine sarcoma cases was high in Razavi Khorasan and North Khorasan, where sarcoma was much more common than adenocarcinoma.

## Discussion

 In 2017, we established the national population-based cancer records for cervical and uterine cancers in terms of incidence rate, geographical distribution by province, growth trend, and histopathological features.

###  Cervical Cancer

 According to global statistics, GLOBOCAN, and World Health Organization (WHO), cervical cancer is the 4th most common cancer in women worldwide, with 604 127 new cases and an ASR of 13.3 in 2020. Eswatini had the highest age-specific incidence rate of 84.5 per 100 000 people, followed by Malawi, Zambia, and other eastern, southern, and middle African countries. On the other hand, Western Asia, including Iran, had the world’s lowest ASR for cervical cancer (ASR 4,1 per 100 000 people). In Asia, the highest ASRs are found in the Maldives (ASR 24.5 per 100 000), Indonesia (ASR 24.4 per 100 000), and Myanmar (ASR 22.6 per 100 000). The three countries with the lowest ASRs (less than 3 per 100 000) were Iraq, Iran, and Yemen. According to the WHO - Cervical Cancer Country Profiles, 2021, Iran had an ASR of 2.3 per 100 000women in 2020.^6,11^ A study by Arab et al in 2014 found that among 428 Iranian women with cervical cancer, ASR was 1.6 per 100 000in 2004, while ages 60–64 had the highest ASR with 6.8.^12^ Chaichian et al in 2018 conducted a retrospective study from 2003 to 2009 with 4273 cervical cancers registered in the National Cancer Registry and Center for Disease Control (CDC) of the Ministry of Health. The incidence of cervical cancer rose from 1.64 in 2003 to 2.17 in 2009. In 2009, the highest cervical cancer incidence rates were 7.4 in Yazd, 5.9 in Isfahan, and 5.84 in Tehran; meanwhile, the lowest rate pertained to Kohkiluye-Boyer Ahmad.^13^

 In our study, the ASR for cervical cancer was 0.99, considerably lower than other populations. It could be due to religious beliefs and rules in Iran that prohibit people from having multiple sex partners.^14^ On the other hand, these beliefs may act as a barrier to seeking medical care, leading to an underestimation of the incidence of cervical and uterine cancer. The highest rates of incidence among provinces belonged to Hormozgan, Yazd, Golestan, and Semnan, respectively, while Bushehr and Ghazvin had the lowest incidence. A study by Talaiezadeh et al in 2013 based on Khuzestan population-based registry reported that the incidence of cervical cancer was 2.56 in the period 2002–2009; however, we found this rate to be 1.32.^15^ Taheri et al in 2014 found the incidence of cervical cancer in Golestan (based on Golestan population-based cancer registry) to be 4.97. This rate is significantly higher compared to our result (1.59).^16^ In 2009, Babaei et al, in a 2-year interval from 2004 to 2006 in Ardabil, found that the incidence of cancer was 1.4 which is almost similar to ours (1.14).^17^ Razavi Khorasan, Tehran and Isfahan were at the top of the list in most studies while Kohkiloye-Boyer Ahmad and Sistan-Baluchestan had the lowest incidence. This lower rate in these provinces should not be interpreted as actually low incidence. Economic and educational situation, poorer access to appropriate healthcare, and migration of patients to larger cities seeking for better health care could be reasons for the lower documented ASR for these provinces compared to others. A study in 2020 ranked Iranian provinces based on their healthcare infrastructures where Kohkiloye-Boyer Ahmad was the 31th province while Tehran, Fars, Razavi Khorasan and Isfahan were the first to fourth.^18^

 In 2020, Amin et al investigated cervical cancer screening disparities across provinces. They found Isfahan to have the highest participants rate with 61%, while Sistan-Bluchestan had the lowest with 7.6%.^19^

 Although all these incidence rates were considerably lower than the majority of the countries around the world, the increasing pattern can be important for scientists and the government in terms of designing efficient implementation programs. As a result, more research is needed to clarify this point in the Iranian population.^10^ Another reason can be the difference between population-based and pathology-based studies. Khorasanizadeh et al in 2013 showed almost a two-fold difference between a pathology-based (ASR = 2.5) and population-based (ASR = 6) cancer registry. A pathology-based study only reports pathology department documents from the entire country, while a population-based approach collects data from many sources, including hospital archives, clinics, and mortality registries.^20^ More than 82% of cervical cancers were confirmed by pathological criteria in this study, which is higher than previous studies and strengthens our findings.

 According to Chaichian et al, the cervical cancer incidence rate in Iran reaches a maximum of 85 and then declines.^13^ In our study, we discovered two age incidence peaks for cervical cancer. These were the age groups of 60–64 and 75–79 years. The decrease in the over 85-year-old age group could be attributed to lack of patient referral to diagnostic centers, lack of access to diagnostic facilities for the elderly, or diagnostic tests performed by doctors at these ages. Regarding the histopathological features, Mortazavi et al indicated that 87% of cervical cancer cases were squamous cell carcinoma, with the remainder being adenosquamous and adenocarcinomas.^21^ Sadeghi et al also reported 73% squamous cell carcinoma, 7.6% adenocarcinoma, 11.5% poorly differentiated carcinoma, and 7.6% carcinoma *in situ* (CIN III).^22^ According to our findings, squamous cell carcinoma was associated with the highest proportion of cervical cancer (approximately 58%), confirming previous reports. However, this was lower than previous studies, indicating an increase in other subtypes of cervical cancer in Iran.

###  Uterine Cancer

 Uterine cancer is the most common female reproductive system malignancy with an ASR of 8.7, according to GLOBOCAN and the Global Cancer Observatory 2020.^6^ WHO in 2020 estimated 1535 new cases of uterine cancer (corpus uteri) with an average ASR of 3.5 per 100 000 women, which places Iran 131st in the world and as one of the low-incidence countries for this cancer.^23^ Uterine cancer had an ASR of 1.7 per 100 000 people in 2008. According to the Iranian Society of Clinical Oncology, the ASR increased to 2.8 per 100 000 people in 2013. In 2017, we found an ASR of 3.29 per 100 000. As a result, the incidence of uterine cancer rises over time in Iran. According to Rezaianzadeh et al, although the incidence rate of uterine cancer is low among Iranians, the associated risk factors such as older age, obesity, polycystic ovarian syndrome, HPV infection, and positive family history are all on the rise, which could lead to an increase in the number of patients in the future.^24^

 The incidence of uterine cancer increases with age; in our study, the age group of 65–69 years had the highest incidence rate of uterine cancer (ASR 15.3 per 100 000 people). In another survey of Egyptian cases of uterine cancer, Alshahrani et al found that the peak incidence was between the ages of 60 and 69.^25^ Furthermore, in the United Kingdom, the peak incidence occurred between the ages of 75 and 79.^26^ This discrepancy can be explained by the fact that life expectancy is not the same in various countries. Also, depending on a country’s healthcare services, earlier screening and diagnostic methods, this range could vary, and the incidence rates can differ.

 There are no accurate statistics on the frequency of uterine cancer diagnosis by pathological features or other methods. Still, pathological confirmation is the most precise way for cancer diagnosis.^27^ However, because imaging and clinical characteristics cannot distinguish sarcoma from leiomyoma, uterine sarcomas are frequently diagnosed only after a surgical procedure for a presumed benign mass.^28^ In the present study, more than 83% of uterine cancer cases were confirmed by pathological features. In other words, pathological studies confirmed more than 90% of patients in Isfahan, Zanjan, Ghazvin, Lorestan, and Yazd. In Bushehr, however, only 30% of cases were pathologically confirmed.

 Most corpus uteri cancers are adenocarcinomas, with endometrioid cancer being the most common type.^29^ This finding was confirmed by our research. In the current study, adenocarcinoma of the uterus accounted for approximately 53% of all uterine cancers. In this study, Ghazvin had the highest percentage of uterine adenocarcinoma, while Razavi Khorasan had the lowest incidence. On the other hand, uterine sarcomas are a rare heterogeneous group of tumors of mesenchymal origin that account for approximately 8% of uterine malignancies, despite previously accounting for only 2% to 3% of all uterine tumors. This increased incidence could be attributed to improved diagnosis as well as an aging population.^30^ In our study, the average incidence of uterine sarcoma in 31 Iranian provinces was 16.35%, more than twice the global average. The high proportion of uterine sarcoma among all types of uterine cancers in Razavi Khorasan can be a serious warning in Iranian cancer epidemiology.

 Our study had several limitations that are pointed out below. First, tumor grades and anatomic sites are not recorded in INPCR and were not evaluated in this study. Second, we did not follow patients to determine the prognosis of cervical and uterine cancers, which is a significant limitation of this study. Also, we did not evaluate the risk factors associated with these cancers. Future studies are needed to determine the potential factors related to the increasing trend of cervical and uterine cancers in Iran.

 In conclusion, our results showed that Iran is a low-risk area for the incidence of cervical and uterine cancers. In this study, the highest rate of cervical cancer was related to squamous cell carcinoma, confirming previous reports. However, this rate was lower than previous studies and suggested an increase in other types of cervical cancer in Iran. In addition, uterine adenocarcinoma was the most common cancer associated with the uteri corpus. Our results also suggested geographical diversities in incidence rates of cancers in different subdivisions of Iran.

## Supplementary Files

Supplementary file 1 contains Tables S1 and S2.
