# Supplementary material for: Uterine and Cervical Cancer in Iran: An epidemiologic analysis of the Iranian National Population-Based Cancer Registry
Source: Arch Iran Med. 2023 Jan 1;26(1):1–7. doi: 10.34172/aim.2023.01 (PMC10685806; doi:10.34172/aim.2023.01)
Supplement: Supplementary file 1 — contains Tables S1 and S2. [file aim-26-1-s001.pdf]

Arch Iran Med. January 2023;26(1):1-S1
10.34172/aim.2023.01

Table S1. histological types of cervical cancers in different provinces

Province	Adenocarcinoma(N)	Adenocarcinoma %	SCC (N)	SCC %	Others (N)	Others %	Unknown (N)	Unknown %
Ardabil	0	0.00	11	73.33	0	0.00	4	26.67
Alborz	6	25.00	11	45.83	4	16.67	3	12.50
Isfahan	11	17.19	36	56.25	7	10.94	10	15.63
Ilam	0	0.00	1	25.00	0	0.00	3	75.00
East.Azarbayjan	5	7.35	40	58.82	14	20.59	9	13.24
West.Azarbayjan	6	13.04	31	67.39	1	2.17	8	17.39
Busher	0	0.00	2	100.00	0	0.00	0	0.00
Tehran	23	14.29	100	62.11	16	9.94	22	13.66
Chaharmahal.Bakhteiari	3	42.86	3	42.86	1	14.29	0	0.00
Razavi.Khorasan	6	7.14	29	34.52	5	5.95	44	52.38
Khoozestan	7	13.46	39	75.00	2	3.85	4	7.69
Zanjan	3	42.86	3	42.86	1	14.29	0	0.00
Semnan	3	25.00	3	25.00	3	25.00	3	25.00
Sistan.Baloochestan	0	0.00	4	66.67	1	16.67	1	16.67
Fars	5	19.23	15	57.69	2	7.69	4	15.38
Ghazvin	0	0.00	4	100.00	0	0.00	0	0.00
Qom	1	11.11	5	55.56	0	0.00	3	33.33
Kordestan	1	10.00	6	60.00	2	20.00	1	10.00
Kerman	3	10.34	19	65.52	1	3.45	6	20.69
Kermanshah	2	15.38	6	46.15	1	7.69	4	30.77
Kohkilooye.Boyerahmad	0	0.00	2	100.00	0	0.00	0	0.00
Golestan	4	13.79	21	72.41	1	3.45	3	10.34
Guilan	1	2.63	19	50.00	1	2.63	17	44.74
Lorestan	0	0.00	4	50.00	3	37.50	1	12.50
Mazandaran	6	14.29	23	54.76	5	11.90	8	19.05
Markazi	1	9.09	6	54.55	1	9.09	3	27.27
Hormozgan	0	0.00	10	40.00	1	4.00	14	56.00
Hamedan	1	7.69	10	76.92	1	7.69	1	7.69
Yazd	2	11.76	14	82.35	1	5.88	0	0.00
South.Khorasan	0	0.00	5	83.33	0	0.00	1	16.67
North.Khorasan	1	12.50	4	50.00	1	12.50	2	25.00

Table S2. histological types of uterine cancers in different provinces

Province	Adenocarcinoma(N)	Adenocarcinoma(%)	Sarcoma(N)	Sarcoma(N)	Others (N)	Other(%)	Unknown(N)	Unk
Ardabil	12	54.55	3	13.64	2	9.09	5	[illegible]
Alborz	47	61.84	11	14.47	8	10.53	10	[illegible]
Isfahan	141	68.45	28	13.59	18	8.74	19	[illegible]
Ilam	3	23.08	2	15.38	3	23.08	5	[illegible]
East.Azarbayjan	74	56.49	19	14.50	14	10.69	24	[illegible]
West.Azarbayjan	42	68.85	5	8.20	3	4.92	11	[illegible]
Busher	4	30.77	0	0.00	0	0.00	9	[illegible]
Tehran	397	68.33	69	11.88	40	6.88	75	[illegible]
Chaharmahal.Bakhteiari	6	50.00	2	16.67	2	16.67	2	[illegible]
Razavi.Khorasan	67	14.69	298	65.35	7	1.54	84	[illegible]
Khoozestan	75	54.35	24	17.39	9	6.52	30	[illegible]
Zanjan	7	70.00	0	0.00	3	30.00	0	[illegible]
Semnan	15	55.56	4	14.81	2	7.41	6	[illegible]
Sistan.Baloochestan	7	43.75	2	12.50	0	0.00	7	[illegible]
Fars	98	57.99	21	12.43	22	13.02	28	[illegible]
Ghazvin	13	81.25	2	12.50	0	0.00	1	[illegible]
Qom	17	48.57	0	0.00	3	8.57	15	[illegible]
Kordestan	10	43.48	3	13.04	3	13.04	7	[illegible]
Kerman	55	68.75	8	10.00	5	6.25	12	[illegible]
Kermanshah	23	67.65	3	8.82	2	5.88	6	[illegible]
Kohkilooye.Boyerahmad	2	50.00	1	25.00	0	0.00	1	[illegible]
Golestan	32	72.73	2	4.55	3	6.82	7	[illegible]
Guilan	64	54.24	13	11.02	7	5.93	34	[illegible]
Lorestan	4	26.67	5	33.33	5	33.33	1	[illegible]
Mazandaran	87	60.00	9	6.21	11	7.59	38	[illegible]

Markazi	16	59.26	0	0.00	2	7.41	9
Hormozgan	10	38.46	7	26.92	2	7.69	7
Hamedan	24	61.54	9	23.08	2	5.13	4
Yazd	36	72.00	3	6.00	7	14.00	4
South.Khorasan	5	33.33	5	33.33	1	6.67	4
North.Khorasan	6	16.22	23	62.16	2	5.41	6
